# MiR-599 Protects Cardiomyocytes against Oxidative Stress-Induced Pyroptosis

**DOI:** 10.1155/2021/3287053

**Published:** 2021-02-18

**Authors:** Xiaoying Fan, Enbo Zhan, Yuan Yao, Ruoxi Zhang, Yong Sun, Xuefeng Tian

**Affiliations:** ^1^Department of Cardiology, Key Laboratories of Education Ministry for Myocardial Ischemia Mechanism and Treatment, 2nd Affiliated Hospital of Harbin Medical University, Harbin 150086, China; ^2^Department of Cardiology, Heilongjiang Provincial People's Hospital, Harbin 150086, China

## Abstract

Oxidative stress is a crucial factor and key promoter of a variety of cardiovascular diseases associated with cardiomyocyte injury. Emerging literatures suggest that pyroptosis plays a key role in cardiac damages. However, whether pyroptosis contributes to cardiomyocyte injury under oxidative stress and the underlying molecular mechanisms are totally unclear. This study was designed to investigate the potential role of pyroptosis in H_2_O_2_-induced cardiomyocyte injury and to elucidate the potential mechanisms. Primary cardiomyocytes from neonatal Wistar rats were utilized. These myocytes were treated with different concentrations of H_2_O_2_ (25, 50, and 100 *μ*M) for 24 h to induce oxidative injury. Our results indicated that mRNA and protein levels of ASC were remarkably upregulated and caspase-1 was activated. Moreover, the expressions of inflammatory factors IL-1*β* and IL-18 were also increased. Luciferase assay showed that miR-599 inhibited ASC expression through complementary binding with its 3′UTR. MiR-599 expression was substantially reduced in H_2_O_2_-treated cardiomyocytes. Upregulation of miR-599 inhibited cardiomyocyte pyroptosis under oxidative stress, and opposite results were found by decreasing the expression of miR-599. Consistently, miR-599 overexpression ameliorated cardiomyocyte injury caused by H_2_O_2_. Therefore, miR-599 could be a promising therapeutic approach for the management of cardiac injury under oxidative condition.

## 1. Introduction

Cardiovascular disease is a pressing worldwide public health problem and the main cause of death and birth defects all over the world [1]. Oxidative stress is involved in the initiation and progression of numerous cardiovascular diseases including cardiac hypertrophy, heart failure, hypertension, and atherosclerosis [2, 3]. Furthermore, considerable research has been conducted to explore antioxidants that can reduce oxidative stress in order to ameliorate cardiovascular diseases [4, 5]. However, the underlying mechanisms of pathophysiological elevated ROS in the cardiovascular system are still not completely revealed [6].

Pyroptosis, a proinflammatory programmed cell death quite different from apoptosis and necrosis, leads to cytokine release that activates proinflammatory immune cell mediators [7]. Inflammasomes are multimeric protein complexes that assemble in the cytosol after sensing pathogen-associated molecular patterns (PAMPs) and danger-associated molecular patterns (DAMPs). Nucleotide-binding oligomerization domain-like receptor family 3 (NLRP3) is a cytosolic receptor activated by PAMPs and DAMPs [8]. The N-terminal pyrin domain (PYD) of NLRP3 serves as a scaffold to nucleate apoptosis-associated speck-like protein containing a CARD (ASC) [9]. Then, pro-caspase-1 is recruited to form an inflammasome. Pro-caspase-1 induces autoproteolytic cleavage into activated cleaved caspase-1 (C-caspase-1). Active caspase-1 cleaves pro-IL-1*β* and pro-IL-18 into biologically activated IL-1*β* and IL-18, respectively [10, 11]. Several studies have provided evidence that pyroptosis is involved in the inflammation process of cardiovascular diseases [12–14]. However, the role of pyroptosis in cardiomyocytes under oxidative stress is totally unclear. Additionally, the potential relationship and pathological mechanisms between oxidative stress and pyroptosis in cardiomyocytes remain to be investigated.

It has been well recognized that microRNAs (miRNAs) play central roles in regulating some key protein-coding genes related to cardiovascular diseases [15]. In this study, we used TargetScan and http://microRNA.org/ to predict the putative complementary miRNAs of ASC. Among them, miR-599 is the most reducing miRNA after H_2_O_2_ treatment. MiR-599 has been identified as an important tumor suppressor gene in multiple studies [16]. However, its role in cardiovascular diseases is not clear.

Taken together, in this study, we investigated the roles of miR-599 and pyroptosis in cardiomyocytes under oxidative stress. Furthermore, the interaction between miR-599 and ASC in the regulation of cardiomyocyte oxidative stress and the possible mechanism was also revealed.

## 2. Methods

### 2.1. Ethics Statement

The study was approved by the Ethics Committee of Harbin Medical University. Experimental procedures were performed in accordance with the recommendations of the Guide for the Care and Use of Laboratory Animals, published by the US National Institutes of Health (NIH Publication No. 85–23, revised 1996).

### 2.2. Cell Culture

Primary cardiomyocytes were taken from 1- to 3-day-old neonatal Wistar rats using collagenase, as described previously [17, 18]. Neonatal rats were sterilized with 75% ethanol, then sacrificed by decapitation. Hearts were isolated and digested by collagenase. Dispersed cells were suspended in total DMEM culture medium and centrifuged. Pooled cells were plated into culture flasks. Bromodeoxyuridine was added into the medium to deplete nonmyocytes. Cardiomyocytes were incubated at 37°C with 5% CO_2_. The cells were treated with different concentrations of H_2_O_2_ (25, 50, and 100 *μ*M) for 24 h.

### 2.3. Gene Transfection

The miR-599 mimic, AMO-599, and its negative control (NC) were synthesized by Guangzhou Ribo Bio Co., Ltd. (Guangzhou, China). The primary cardiomyocytes were pretreated by 100 *μ*M H_2_O_2_ for 24 h. Then, transfection of miR-599 mimic, AMO-miR-599, and NC using X-treme GENE siRNA Transfection Reagent (catalog no. 04476093001; Roche, Mannheim, Germany) according to the manufacturer's instructions.

### 2.4. Cell Viability Assay

3-(4,5-dimethylthiazol-2-yl)-2,5-diphenyltetrazolium bromide (MTT) assay was performed to evaluate cell viability of different groups. We followed the methods of Zhang et al. 2017 [17, 18]. Briefly, cells were seeded in 96-well plates followed by gene transfection or H_2_O_2_ treatment for 24 h. 20 *μ*L MTT solution was added into cell culture medium for 4 h. Then, 150 *μ*L DMSO was added to dissolve the formazan crystals. Absorbance was measured at 570 nm on a plate reader.

### 2.5. RNA Extraction and Real-Time PCR

Total RNAs from cells were extracted using 1 mL of Trizol reagent (Invitrogen) according to the manufacturer's instructions. cDNA synthesis was performed using the High Capacity cDNA Reverse Transcription Kit (Applied Biosystems, Carlsbad, CA, USA, Cat. no. 4368814). The SYBR Green PCR Master Mix Kit (Applied Biosystems, Cat. no.4309155) was used to quantify the relative mRNA levels of miR-599, ASC, IL-18, and IL-1*β*. Real-time PCR was performed with the 7500 FAST Real-Time PCR System (Applied Biosystems) for 40 cycles, with GAPDH and U6 serving as internal controls. The sequences of primer pairs are as follows: miR-599: Forward, 5′-GTTGTGTCAGTTTA-3′, Reverse, 5′-CAGTGCGTGTCGTGGAGT-3′; miR-122: Forward, 5′-TGGAGTGTGACAATGG-3′, Reverse, 5 ′-CAGTGCGTGTCGTGGAGT-3 ′; miR-383: Forward, 5 ′-CGCGCAGATCAGAAGGTGA-3 ′, Reverse, 5 ′-AGTGCAGGGTCCGAGGTATT-3 ′; ASC: Forward, 5′-TGTGGCTACTGCAACCAGTG-3′, Reverse, 5′-TTGGTTGGTGGTCTCTGCAC-3′; IL-1*β*: Forward, 5′-TGTGATGAAAGACGGCACAC-3′, Reverse, 5′-CTTCTTCTTTGGGTATTGTTTGG-3′; IL-18: Forward, 5′-ACCGCAGTAATACGGAGCAT-3′, Reverse, 5′-CGTTGGCTGTTCGGTCGATA-3′.

### 2.6. Western Blotting

The protein isolation was performed as previously described [17]. Protein samples of 100 *μ*g/well were loaded in a 10% SDS polyacrylamide gel and transferred onto a nitrocellulose filter membrane, then blocked by 5% nonfat milk dissolved in PBS for 2 h. The membrane was probed with primary antibodies against ASC (Abcam), caspase-1 (Abcam), and C-caspase-1 (Abcam). GAPDH (Zhongshanjinqiao, Inc., Beijing, China) was used as an internal control. Western blot bands were quantified by the Odyssey Infrared Imaging System (LI-COR, Lincoln, NE, USA) by measuring band intensity (Area × OD).

### 2.7. Enzyme-Linked Immunosorbent Assay

Cell contents of IL-1*β* (USCN, Wuhan, China) and IL-18 (USCN, Wuhan, China) were determined by using ELISA kits following the manufacturer's instructions.

### 2.8. Bioinformatic Methods for Targetscan and microrna.org

http://www.targetscan.org/ and http://www.microrna.org/ were used to predict the complementary binding miRNAs with ASC 3′UTR. After getting the 3′UTR region of the ASC in NCBI, targetscan and http://microrna.org/ were used to predict potential miRNAs that may be able to bind to 3′UTR of ASC (>5mer).

### 2.9. Dual-Luciferase Gene Reporter Assay

For the dual-luciferase gene reporter assay between ASC and miR-599, the full length of wild-type ASC was amplified by PCR, and then, the PCR products were subcloned into psiCHECK-2 luciferase reporter vector (Luc-ASC-WT; Promega, WI, USA). Luc-miR599 mutant and Luc-miR599 WT was also constructed. Forty-eight hours later, Renilla and firefly luciferase activities were measured with the Dual-Luciferase Reporter Assay System (Roche, Mannheim, Germany) and GloMax Luminometry System (Promega, WI, USA).

### 2.10. Statistical Analysis

The data are shown as the mean ± S.E.M. Differences among multiple groups were analyzed using one-way ANOVA followed by Bonferroni's multiple-comparisons test. Two-tailed Student's *t*-test was used for comparison between two groups. Only *p* < 0.05 was considered statistically significant. Data are analyzed by GraphPad Prism version 5.0.

## 3. Results

### 3.1. H_2_O_2_ Induces Pyroptosis in Cardiomyocytes

**Figure 1:**
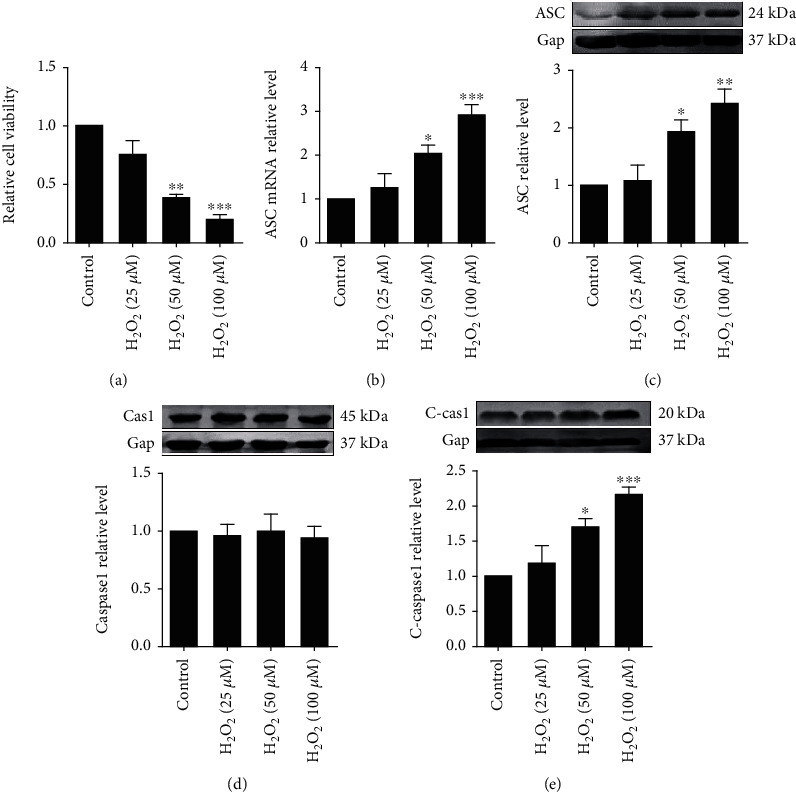
H_2_O_2_ induces pyroptosis in cardiomyocytes. (a) Exposure of neonatal rat cardiomyocytes to different concentrations of H_2_O_2_ (0, 25, 50, 100 *μ*M) for 24 h. Cell viability was determined by MTT assay. (b) ASC mRNA levels determined by qRT-PCR. (c) ASC protein levels determined by Western blot analysis. Caspase-1 (d) and cleaved-caspase-1 (C-caspase-1) (e) protein levels determined by Western blot analysis. The data are presented as mean ± SEM of five independent experiments. ^∗^*p* < 0.05, ^∗∗^*p* < 0.01, ^∗∗∗^*p* < 0.001, vs. Control.

**Figure 2:**
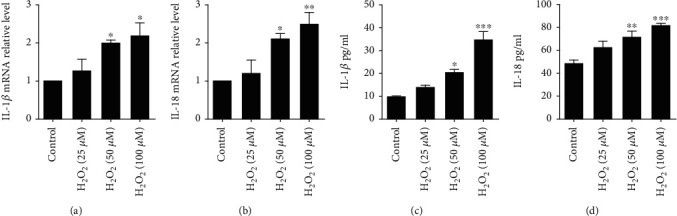
H_2_O_2_ upregulates IL-1*β* and IL-18 expression in cardiomyocytes. QRT-PCR analysis of IL-1*β* (a) and IL-18 (b) mRNA levels after H_2_O_2_ treatment. IL-1*β* (c) and IL-18 (d) concentration in cell culture medium determined by ELISA analysis. The data are presented as mean ± SEM of five independent experiments. ^∗^*p* < 0.05, ^∗∗^*p* < 0.01, ^∗∗∗^*p* < 0.001, vs. Control.

In order to study the effect of H_2_O_2_ on cardiomyocytes, cell viability was measured after different concentration of H_2_O_2_ treatment. Consistent with other studies [19], H_2_O_2_ reduced cardiomyocyte viability in a concentration-dependent manner, with statistical significance at 50 and 100 *μ*M of H_2_O_2_, respectively (Figure 1(a)). To explore whether H_2_O_2_ could induce pyroptosis, we examined ASC expression in primary cardiomyocytes under different concentration of H_2_O_2_. The results showed that the mRNA and protein levels of ASC were significantly upregulated, especially under higher H_2_O_2_ concentration (Figures 1(b) and 1(c)). Moreover, pro-caspase-1 was recruited by ASC to form inflammasome, which was responsible for pyroptosis initiation. Our results revealed that cleaved caspase-1 was also increased by H_2_O_2_ incubation (Figures 1(d) and 1(e)). These results indicated an induction of cardiomyocyte pyroptosis by H_2_O_2_. Furthermore, pyroptosis of cardiomyocytes was substantially enhanced by H_2_O_2_ along with consistently observed increasing in IL-1*β* and IL-18 (Figures 2(a)–2(d)).

### 3.2. MiR-599 Is Downregulated in Cardiomyocytes under Oxidative Stress

**Figure 3:**
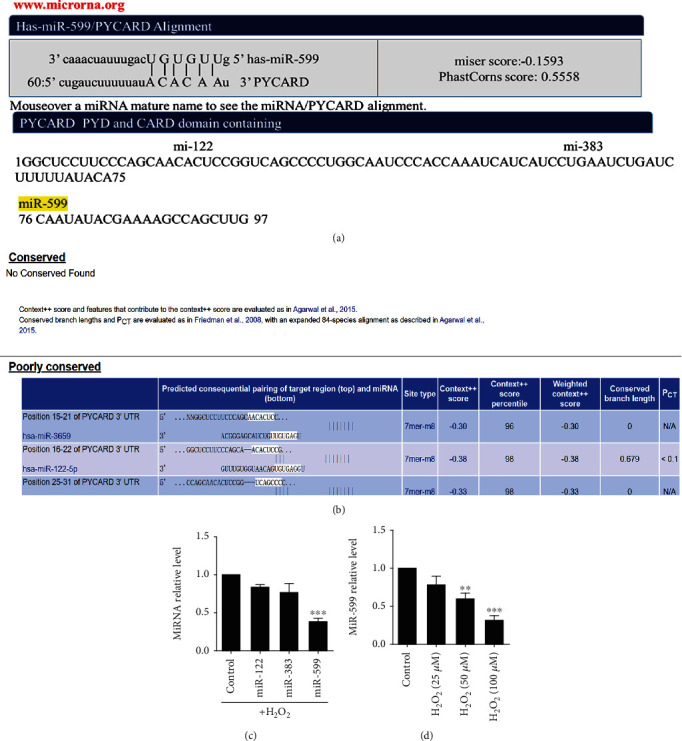
MiR-599 is remarkably inhibited by H2O2 treatment. Sequence complementarity between miRNAs and ASC (PYCARD) predicted by http://MicroRNA.org/ (a) and TargetScan (b) databases. (c) QRT-PCR analysis of miR-122, miR-383, and miR-599 expression levels after H2O2 treatment. The data are presented as mean ± SEM of five independent experiments. ^∗∗^*p* < 0.01, ^∗∗∗^*p* < 0.001, vs. Control.

We further investigated the possible involvement of miRNAs in H_2_O_2_-induced cardiomyocyte pyroptosis. As ASC was remarkably increased in cardiomyocytes treated by H_2_O_2_, http://MicroRNA.org/ and Targetscan databases were used to predict the complementary binding miRNAs with ASC 3′UTR. We found that miR-122, miR-383, and miR-599 were the potential upstream regulator of ASC as predicted by http://MicroRNA.org/ (Figure 3(a)). However, there was no conserved predicted miRNA in Targetscan database (Figure 3(b)). In 100 *μ*M of H_2_O_2_-treated cardiomyocytes, expression levels of three miRNAs were tested by qRT-PCR. The results suggested that miR-599 demonstrated the most pronounced downregulation in the oxidative condition (Figure 3(c)), and H_2_O_2_ treatment decreased miR-599 expression in a dose-dependent manner (Figure 3(d)). And we noticed that inhibition of miR-599 alone did not induce the pyroptosis of cardiomyocytes (Supplemental Fig. 1A, B).

### 3.3. MiR-599 Directly Targets ASC

**Figure 4:**
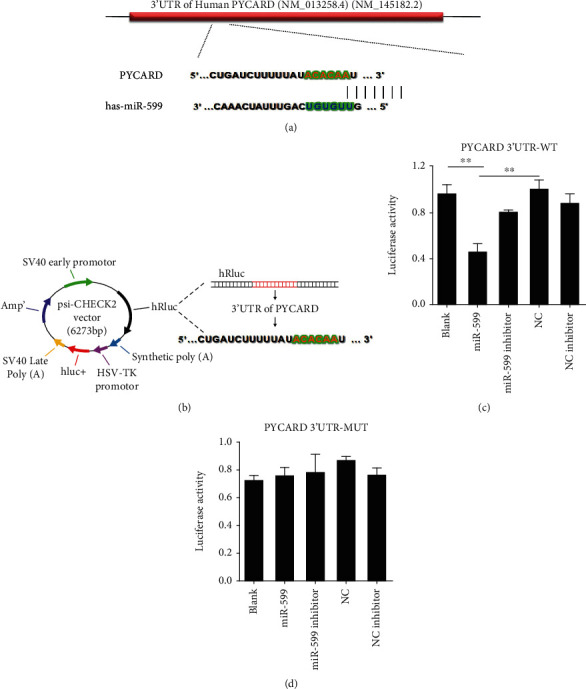
Identification of ASC as a direct target of miR-599. (a) Sequence complementarity between miR-599 and ASC. The letters in green indicate matched bases. (b) Luciferase reporter constructs containing 3′UTR sequence of PYCARD. Luciferase activities with wild-type (WT) constructed plasmid (c) or mutant (MUT) 3′UTR sequences of PYCARD (d). Data are expressed as mean ± SEM of three independent experiments. ^∗∗^*p* < 0.01 vs. miR-599. Negative control (NC); NC is the negative control of miRNA-599, and NC inhibitor is the negative control of miRNA-599 inhibitor.

We next performed a series of functional studies to determine the link between miR-599 and ASC. Computational analysis predicted a conserved binding site for miR-599 in the 3′-UTR of ASC gene (PYCARD) (Figure 4(a)). To verify that miR-599 directly targets ASC, we prepared luciferase reporter carrying the PYCARD 3′-UTR (Figure 4(b)). The sequence of the PYCARD 3′UTR mutation was available in supplemental Fig 1D. Cotransfection of miR-599 with the luciferase reporter vector into HEK293 cells caused a sharp decrease in luciferase activity compared with transfection of the luciferase vector alone. The miR-599-induced depression of luciferase activity was rescued by an antisense inhibitor oligonucleotide (miR-599 inhibitor) used to knockdown miR-599 (Figure 4(c)). However, miR-599 failed to affect the luciferase activity elicited by the construct carrying the mutant ASC 3′-UTR fragment (Figure 4(d)). The data indicated that miR-599 inhibited PYCARD translation through complementary binding to its 3′UTR.

### 3.4. MiR-599 Prevents Cardiomyocyte Pyroptosis under Oxidative Stress by Targeting ASC

**Figure 5:**
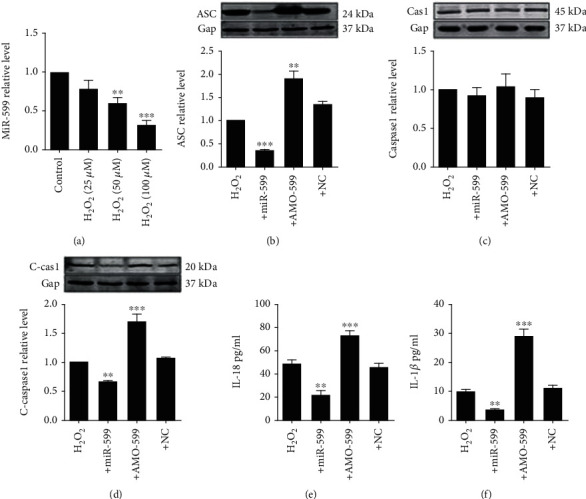
Overexpression of miR-599 inhibits H2O2 induced pyroptosis by directly targeting ASC. (a) QRT-PCR analysis of ASC mRNA levels after miR-599 mimic and inhibitor transfection. (b) Western blot analysis of ASC protein levels. Western blot analysis of caspase-1 (c) and C-caspase-1 (d) protein levels. IL-1*β* (e) and IL-18 (f) concentration in cell culture medium determined by ELISA analysis. The data are presented as mean ± SEM of five independent experiments. ^∗^*p* < 0.05, ^∗∗^*p* < 0.01, ^∗∗∗^*p* < 0.001, vs. NC.

Next, we wondered if increasing miR-599 expression would have prevented cardiomyocyte pyroptosis. SATB2 and TGFB2 are well-known targets for miR-599; the results shown that miR-599 was transfected effectively (Supplemental Fig. 1C). We found that transient upregulation of miR-599 inhibited ASC expression in genetic and protein levels, which was reversed by transfection with complementary inhibitory sequence of miR-599 (AMO-599), indicating that knockdown of miR-599 improved ASC expression (Figures 5(a) and 5(b)). Meanwhile, caspase-1 activation was also suppressed by miR-599 overexpression, while increased by AMO-599 transfection (Figures 5(c) and 5(d)). Moreover, we evaluated the effects of miR-599 on the levels of inflammatory factors IL-1*β* and IL-18 using ELISA. Forced expression of miR-599 significantly decreased IL-1*β* and IL-18 levels, which were reversed by transfection with AMO-599 in 100 *μ*M of H_2_O_2_-treated cardiomyocytes (Figures 5(e) and 5(f)). These data suggested that miR-599 inhibited H_2_O_2_-induced pyroptosis through downregulating the expression of ASC as well as the downstream signaling factors.

### 3.5. MiR-599 Protects Cardiomyocytes from Oxidative Injury

**Figure 6:**
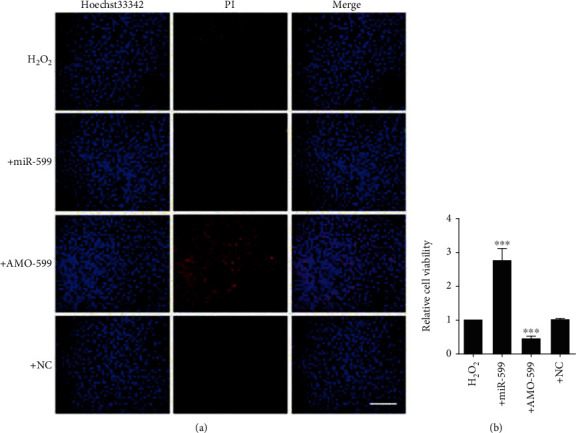
MiR-599 protects cardiomyocytes from oxidative injury. (a) Photomicrographs of double-fluorescent staining with PI (red) and Hoechst33342 (blue). Scale bar indicates 100 *μ*m. (b) Cell viability was determined by MTT assay. The data are presented as mean ± SEM of five independent experiments. ^∗∗∗^*p* < 0.001 vs. NC.

PI/Hoechst33342 staining further confirmed that transfection of miR-599 inhibited H_2_O_2_-induced cardiomyocyte injury, which was predictably deteriorated by AMO-599 (Figure 6(a)). Consistently, as shown in Figure 6(b), forced transient expression of miR-599 significantly increased cell viability in the presence of H_2_O_2_, and the effect was nearly diminished after transfection with AMO-599. These results demonstrated that miR-599 relieved cardiomyocyte injury induced by oxidative stress and the inhibited pyroptosis might be responsible for the reason.

## 4. Discussion

Oxidative stress-induced cardiac injury is the primary cause of cardiovascular diseases [20–22]. Studies on the underlying mechanisms are very crucial to develop therapeutic strategies and prevent premature cardiac cell loss in patients. Pyroptosis is a highly inflammatory form of programmed cell death and is triggered by ASC-inflammasome formation [11, 23]. In recent years, a tremendous amount of effort has been devoted to discover the mechanisms of pyroptosis in many diseases and to determine the genes and pathways involved in this process [24]. Published evidence indicated that serelaxin attenuated myocardial I/R injury and the subsequent caspase-1 activation via eNOS-dependent mechanism [25]. Huang et al. found that H_2_S suppressed HG-induced cardiomyocyte inflammation and apoptosis by inhibiting the TLR4/NF-*κ*B pathway and its downstream NLRP3 inflammasome activation [26]. Studies from other group also revealed DM-induced arrhythmias could be successfully treated by inhibiting the IL-1*β* axis with either IL-1 receptor antagonist or by inhibiting the NLRP3 inflammasome [27, 28]. In fact, different concentrations of H_2_O_2_ have different effects on cells. More than 200 *μ*M of hydrogen peroxide induces apoptosis in endothelial cells, smooth muscle cells, and epithelial cells [29] [30, 31]. Tong et al. reported that low level of H_2_O_2_ significantly promoted endothelial cell proliferation, migration, and tube formation; the mechanism is related to Nox-derived ROS [32]. And we found that there are no studies confirming that low concentrations of H_2_O_2_ promote the proliferation of smooth muscle cells and epithelial cells.

Reactive oxygen species (ROS) serves as important inflammation activating signals. Recently, several studies have revealed that ROS induces NLRP3 inflammasome-dependent pyroptosis in human keratinocyte HaCaT cells, intestinal epithelial cells, and astroglial cells [24, 33, 34]. Mitogen-activated protein kinases (MAPK) and Extracellular signal-regulated protein kinases 1 and 2 (ERK1/2) signaling pathways were involved in this process [35]. However, whether pyroptosis inflammasome could be induced by oxidative stress in cardiomyocytes is still unknown. Our study is the first study that demonstrated H_2_O_2_ stress brought out pyroptosis in cardiomyocytes as a concentration-dependent manner. ASC mRNA and protein expressions were upregulated, and activated caspase-1 and increased IL-1*β* and IL-18 levels were also observed. We further elucidated the molecular mechanisms involved in these processes.

In just over two decades since the discovery of the first microRNA (miRNA), the field of miRNA biology has expanded considerably. Insights into the roles of miRNAs in development and disease have made miRNAs attractive tools and targets for novel therapeutic approaches [15]. The regulatory link between oxidative stress and pyroptosis in cardiomyocytes remained to be discovered. Whether miRNAs responsible for this setting remains unclear, although some microRNAs have been identified that are regulated by oxidative stress that modulate cardiovascular physiopathology [36]. Here, we firstly identified a directly inhibitory miRNA of ASC, miR-599, using luciferase reporter assay. miR-599 was a well-characterized tumor-suppressor that regulated tumor cell proliferation, migration, and invasion. Periostin, SATB2, TGFB2, and MYC have been proved to be its targets to data [37–39]. Its role in cardiovascular diseases still remains unclear. Our study showed that miR-599 was downregulated in primary cardiomyocytes after 50 and 100 *μ*M of H_2_O_2_ treatment for 24 h. Moreover, gain-of-function as well as loss-of-function experiments further confirmed the antipyroptotic effects of miR-599. Meanwhile, PI/Hoechst staining and cell viability analysis suggested that overexpression of miR-599 ameliorated cardiomyocyte injury caused by H_2_O_2_ treatment, whereas miR-599 inhibition contributed to oxidative-induced cardiomyocyte injury.

## 5. Conclusions

The present study provided three novel findings: (1) the significant contribution of pyroptosis to oxidative stress-induced cardiomyocytes injury, (2) the antipyroptotic property of miR-599 by directly targeting ASC, and (3) the cardiac protective effects of miR-599 under oxidative stress, while the AMO-599 aggravated cardiac damages. These findings will help to gain knowledge about the molecular mechanisms of cardiomyocyte injury during oxidative stress and show that miRNA-based approaches may contribute to the development of more effective antioxidant therapies.

## Data Availability

The original data of our experiment has been send separately. The result of immunofluorescence is edited as PDF version, and Western blot is edited as DOC version. Especially, all the experimental data could be open by GraphPad Prism 5 application.

## Supplementary Materials

Supplementary Materials Supplemental Figure1: (a) Photomicrographs of double-fluorescent staining with PI (red) and Hoechst33342 (blue). (b) Cell viability was determined by CCK8 assay. (c) SATB2 and TGFB2 protein levels determined by Western blot analysis. (d) The sequence of the PYCARD 3′UTR mutation.
