# Supplementary material for: MiR-599 Protects Cardiomyocytes against Oxidative Stress-Induced Pyroptosis
Source: Biomed Res Int. 2021 Feb 18;2021:3287053. doi: 10.1155/2021/3287053 (PMC7906806; doi:10.1155/2021/3287053)
Supplement: Supplementary Materials — Supplemental Figure1: (a) Photomicrographs of double-fluorescent staining with PI (red) and Hoechst33342 (blue). (b) Cell viability was determined by CCK8 assay. (c) SATB2 and TGFB2 protein levels determined by Western blot analysis. (d) The sequence of the PYCARD 3′UTR mutation. [file 3287053.f1.zip › Supple_MAT.Data.Avil/Western-blot.docx]

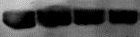


ASC gap


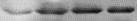


ASC


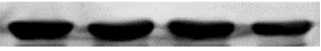


cas 1 gap


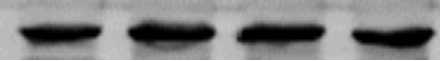


cas1


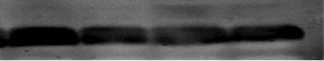


c-cas1 gap


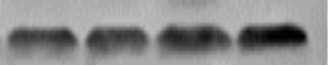


c-cas1


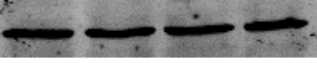


tug ASC gap


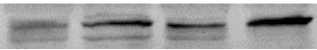


tug ASC


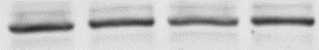


tug caspase1


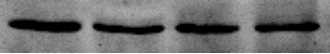


tug c-caspase1 gap


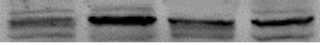


tug c-caspase1


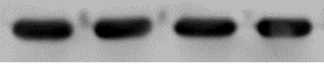


tug1 caspase1 gap
